# The glymphatic–lymphatic axis: known pathways, open questions, and emerging roles in neurodegeneration

**DOI:** 10.1186/s40035-026-00588-8

**Published:** 2026-10-09

**Authors:** Yumeng La, Jin Yuan, Jun Chen, Kejia Lv, Xiaoxi Wang, Qiqi Gao, Yujue Wang, Chen Wang, Bin Xu, Ying Yang, Jing Zhang

**Affiliations:** 1 Department of Pathology, The First Affiliated Hospital, Zhejiang University School of Medicine, Hangzhou, 310003 China; 2 Nanhu Brain-Computer Interface Institute, Hangzhou, 311121 China; 3 Lingang Laboratory, Shanghai, 201103 China; 4 Department of Stomatology, The First Affiliated Hospital, Zhejiang University School of Medicine, Hangzhou, 310003 China; 5 National Human Brain Bank for Health and Disease, Zhejiang University, Hangzhou, 310012 China

**Keywords:** Cerebrospinal fluid, Meningeal lymphatic vessels, Glymphatic system, Immunity, Central-peripheral communication, Neurodegeneration

## Abstract

The discovery of the glymphatic system and meningeal lymphatic vessels has transformed our understanding of brain fluid homeostasis and central–peripheral communication, highlighting the critical role of cerebrospinal fluid in brain clearance. While substantial progress has been made in defining the structural components and physiological regulators of brain clearance, major uncertainties remain regarding (1) how these systems interact as an integrated physiological network, (2) how they fail in disease, and (3) whether they can be therapeutically manipulated. In this review, we first summarize the anatomical and physiological organization of the glymphatic–lymphatic axis, with particular emphasis on its regulatory mechanisms in light of rapid advances in this field. We then discuss how the glymphatic–lymphatic axis functionally integrates with peripheral immune and metabolic systems and describe how impairment of these clearance pathways is associated with the accumulation of pathological proteins, neuroinflammation, and vascular dysfunction and may contribute to the progression of neurodegenerative diseases such as Alzheimer’s and Parkinson’s diseases. Finally, we discuss emerging therapeutic strategies and biomarkers targeting the glymphatic–lymphatic axis. By redefining the central–peripheral crosstalk, the glymphatic–lymphatic axis provides a unifying framework for understanding brain clearance, neurodegeneration, and future therapeutic interventions.

## Background

Unlike most peripheral organs, the brain parenchyma lacks a conventional lymphatic vasculature. Instead, fluid and solute efflux from the central nervous system (CNS) relies on a series of specialized pathways [1]. The glymphatic system has been proposed as a brain-wide pathway contributing to fluid transport and metabolic waste clearance in the CNS. This model centers on the exchange between cerebrospinal fluid (CSF) and interstitial fluid (ISF) along perivascular and parenchymal routes [2].

Specifically, produced primarily by the choroid plexus, CSF circulates through the ventricular system and subarachnoid space and is generally thought to enter the brain parenchyma along perivascular spaces. Following exchange between CSF and ISF, fluid and solutes are cleared through multiple proposed pathways into the venous circulation or through extracranial lymphatic pathways before returning to the peripheral circulation [1–3]. Together, the glymphatic system and these efflux pathways form an integrated glymphatic–lymphatic axis. This axis not only clears metabolic waste but also facilitates immune surveillance, thereby creating a functional interface linking the CNS with peripheral immune and vascular systems [4–7].

Despite these advances, fundamental questions about the glymphatic–lymphatic axis remain. A central unresolved question is whether impaired clearance represents a primary pathogenic driver, a secondary consequence of neurovascular and cellular dysfunction, or a bidirectional amplifier that accelerates disease progression once pathology is established [4, 8–10]. Addressing this question is critical for understanding how alterations in the CSF–ISF dynamics and meningeal lymphatic drainage contribute to neuroinflammation and disease progression.

In this review, we first summarize the anatomical and physiological organization of the glymphatic–lymphatic axis, including its structural components, fluid pathways, and ongoing mechanistic controversies, with particular emphasis on its physiological regulation by mechanical, systemic, and sleep-related factors. We then examine how the glymphatic–lymphatic axis functionally integrates with peripheral immunity and metabolism, highlighting bidirectional CNS–peripheral interactions relevant to brain clearance. Next, we describe how failure of these clearance routes promotes the accumulation of toxic proteins, neuroinflammation, and vascular dysfunction, thereby accelerating neurodegenerative processes such as Alzheimer’s disease (AD) and Parkinson’s disease (PD). Finally, we discuss emerging therapeutic strategies and biomarkers targeting the glymphatic–lymphatic axis. By redefining the CNS–peripheral crosstalk, the glymphatic–lymphatic axis provides a unifying framework for understanding and potentially mitigating neurodegeneration.

## Anatomical and physiological organization of the glymphatic–lymphatic axis

### Structural components and fluid pathways

**Fig. 1:**
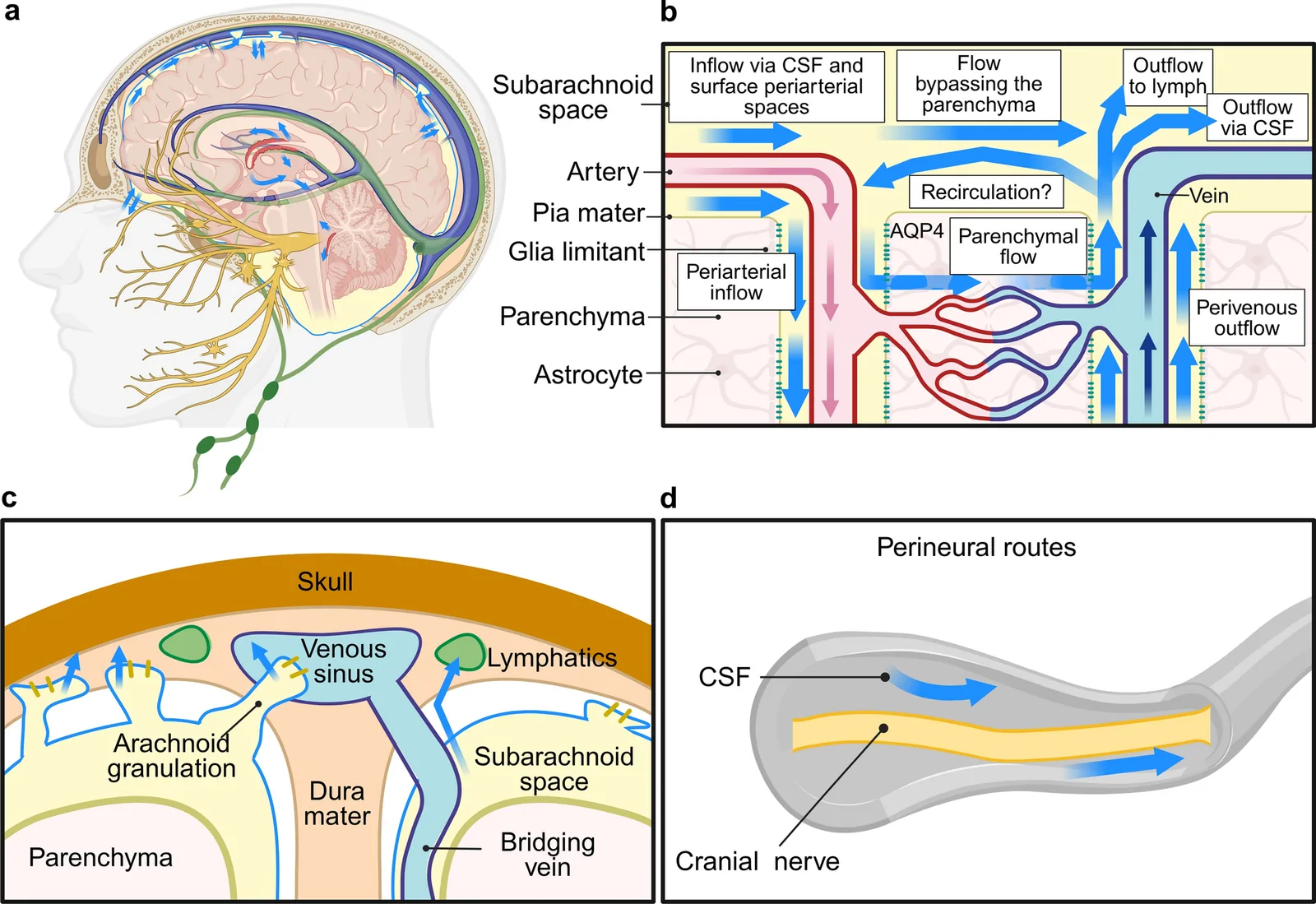
Fluid clearance pathways of the glymphatic–lymphatic axis. **a** Schematic of major CSF circulation and clearance routes in the brain. **b** Schematic illustrating the proposed five-stage glymphatic transport model in the brain parenchyma and the SAS*.* (1) Fluid enters periarterial spaces from the brain surfaces. (2) It translocates into the interstitium, with water movement partially facilitated by AQP4 in astrocytic endfeet adjacent to endothelial cells. (3) Fluid and solutes move through the interstitium toward proposed perivenous efflux routes. (4) Fluid moves into perivenous spaces, with water movement partially facilitated by AQP4. (5) It ultimately exits to the subarachnoid space and downstream lymphatic drainage pathways. Adapted from Hladky and Barrand [16], licensed under CC BY 4.0 (http://creativecommons.org/licenses/by/4.0/). The original figure was redrawn and modified by the authors. **c** Depiction of fluid efflux routes from the SAS to the dural venous sinuses and lymphatics. **d** CSF drainage along perineural spaces. CSF, cerebrospinal fluid; SAS, subarachnoid space; AQP4, aquaporin-4. (Created with BioRender.com)

The glymphatic–lymphatic axis describes a functional continuum linking CSF and intraparenchymal glymphatic transport to extracranial lymphatic drainage. CSF is primarily produced by the choroid plexus within the ventricular system and flows from the lateral ventricles through the third ventricle and cerebral aqueduct into the fourth ventricle before entering the subarachnoid space (SAS) [1, 6, 11–13] **(**Fig. 1a**)**. CSF inflow and outflow pathways, together with their regulatory mechanisms, have been comprehensively reviewed by Hladky et al. [14]. Under physiological conditions, CSF is generally thought to enter the brain parenchyma from the SAS predominantly along periarterial spaces, where it exchanges with ISF, followed by clearance via proposed perivascular and other efflux pathways [14, 15] **(**Fig. 1b**)**. In addition to this periarterial entry route, under pathological conditions such as hydrocephalus, CSF may also be absorbed across the ependymal lining of the ventricles into the brain interstitium [14, 16].

However, the precise mechanism by which CSF exchanges with ISF within the brain parenchyma remains incompletely understood. The original glymphatic hypothesis proposed that the CSF–ISF exchange is driven primarily by convective bulk flow or advection [1], whereas increasing evidence suggests that diffusion or diffusion-dominated dispersion may play a major role [14, 16]. Following exchange with ISF, fluid and solutes may subsequently drain along parenchymal perivascular pathways associated with arteries or veins, cross the pial surface back into the SAS, or move along white-matter tracts toward the ventricular system [14, 16]. Following clearance from the brain parenchyma, fluid can subsequently leave the cranial compartment through multiple proposed CSF outflow pathways, including arachnoid granulations [17, 18], dural venous sinuses [15], dural lymphatic vessels [19], cribriform plate-associated lymphatic routes [20], and spinal outflow pathways [21, 22] (Fig. 1a, c, d).

Rodents generally lack the large, well-developed arachnoid granulations observed in humans, although microscopic arachnoid villi have been identified in rats [23]. In rodents, perineural and lymphatic pathways associated with the cribriform plate and skull base are thought to contribute substantially to CSF outflow and subsequent drainage to cervical lymph nodes [24, 25]. Other potential outflow routes include optic nerve sheath-associated and spinal pathways [6]. Recent studies have also identified a subarachnoid lymphatic-like membrane (SLYM) in mice [26], which has been proposed to separate the SAS into superficial and deep compartments. This membrane may compartmentalize the SAS and influence periarterial CSF influx into the brain parenchyma. However, whether SLYM directly governs CSF flow direction or restricts CSF movement toward specific outflow routes, such as the cribriform plate-associated pathway, remains to be determined. In addition, arachnoid cuff exit (ACE) points have recently been identified as additional sites of CSF–dura exchange, where bridging veins cross the arachnoid barrier and create localized discontinuities that allow CSF to exit the SAS and access the dura [19].

In humans, by contrast, CSF is thought to drain through multiple routes, including arachnoid granulations and villi leading to dural venous sinuses and dural lymphatic vessels, the cribriform plate, optic nerve sheath-associated pathways, and spinal routes, before ultimately returning to the peripheral circulation [6, 15, 22, 27]. Although the cribriform plate-associated route is a major CSF outflow pathway in rodents, its relative contribution in humans remains unclear. Human imaging studies have reported conflicting findings regarding cribriform plate-associated CSF clearance. One intrathecal gadobutrol-enhanced MRI study found marked tracer enrichment in CSF spaces adjacent to the cribriform plate but no significant enrichment in the nasal mucosa [28], whereas dynamic PET studies detected tracer kinetics in the superior nasal turbinates consistent with a potential brain-to-nasal CSF clearance pathway [29, 30]. These discrepancies may be attributable to differences in tracer kinetics, imaging sensitivity, and sampling time points; for example, rapid tracer clearance from the nasal mucosa could reduce tracer concentrations below the detection threshold of MRI, while low-volume CSF efflux may also be underestimated. If the contribution of cribriform plate-mediated CSF outflow is limited in humans, drainage via arachnoid granulations and villi into dural venous sinuses and drainage via dural lymphatic vessels may play more prominent roles, although the quantitative contribution of each pathway requires further investigation.

Together, these anatomical pathways constitute the current structural framework of the glymphatic–lymphatic axis. However, how these pathways are functionally integrated, what drives fluid movement through them, and how individual routes contribute to overall brain clearance remain subjects of active investigation.

### Ongoing controversies and unresolved mechanisms of glymphatic transport

Although the glymphatic model has substantially advanced our understanding of brain fluid transport and waste clearance, several key mechanistic aspects remain under active debate. Importantly, these controversies do not question the existence of brain fluid and solute transport per se, but rather concern the organization, driving forces, and quantitative contributions of different clearance pathways. Recent reviews have emphasized that, although several core features of brain clearance are now broadly supported, important questions regarding paravascular flow direction, transport mechanisms within the parenchyma, and relevance to human disease remain largely unresolved [3, 8, 31]. These unresolved issues highlight the need to further refine current models of perivascular transport within the CNS.

One major debate concerns the directionality of paravascular CSF flow. The classical glymphatic model proposes that CSF enters the brain predominantly along periarterial spaces, exchanges with ISF, and exits via perivascular pathways before reaching downstream lymphatic routes [1]. While this model is supported by numerous tracer-based studies, recent work has questioned whether paravascular transport follows a single, uniform, unidirectional pathway under physiological conditions or instead reflects multiple parallel routes whose relative contributions vary with experimental conditions, anatomical location, and physiological state [14, 16]. In addition, intramural periarterial drainage has been proposed as an alternative pathway for metabolic waste clearance, particularly for the removal of interstitial solutes such as β-amyloid (Aβ) along the basement membranes of cerebral arterial walls [32]. In contrast to the glymphatic system, which emphasizes perivascular CSF–ISF exchange, intramural periarterial drainage describes a clearance route that depends on basement membrane pathways associated with vascular smooth muscle cells [33]. Neuropathological studies of cerebral amyloid angiopathy suggest a potential role of intramural periarterial drainage in amyloid deposition and vascular pathology [34]. Rather than representing mutually exclusive models, these pathways may constitute complementary components of brain waste clearance whose relative importance remains to be established.

Another unresolved question concerns how proposed parenchymal clearance pathways connect anatomically and functionally with downstream meningeal lymphatic drainage. Although these pathways are widely considered components of an integrated brain clearance system, their precise anatomical continuity and relative contributions remain incompletely understood. For example, an apparent spatial discordance exists between the proposed perivenous glymphatic outflow, which is thought to converge toward the dorsal dural venous sinuses, and the observation that functionally active meningeal lymphatic vessels (mLVs) are concentrated predominantly at the ventral skull base. To reconcile this discrepancy, Jacob et al. identified an anterior meningeal lymphatic network surrounding the cavernous sinus that may provide an anatomical link between these two drainage territories, potentially channeling fluid toward the cervical lymph nodes [21]. Nevertheless, whether perivenous drainage and meningeal lymphatic outflow represent sequential components of a continuous clearance pathway or partially independent drainage routes remains unresolved.

A related unresolved issue is the relative contribution of bulk flow and diffusion to solute transport within the brain parenchyma. Although the original glymphatic hypothesis proposed that the CSF–ISF exchange is driven primarily by convective bulk flow, increasing evidence suggests that the solute movement likely reflects a combination of advection, diffusion, and dispersion rather than a single dominant transport mechanism, with their relative contributions varying according to solute size, anatomical compartment, and physiological state [35]. Thus, the extent to which bulk flow contributes to brain clearance remains incompletely understood.

Beyond these debates regarding the physical mechanisms of glymphatic transport, the molecular regulation of CSF–ISF exchange also remains incompletely understood. Astrocytic aquaporin-4 (AQP4) has been proposed as an important contributor to glymphatic transport, although its precise role remains controversial. As a water-selective membrane channel, AQP4 facilitates transmembrane water movement rather than directly transporting solutes. Despite not providing an explanation for solute transport, numerous studies continue to support an important role for AQP4 in CSF–ISF exchange [1, 36]. Notably, AQP4 deficiency has been reported to reduce, but not abolish, CSF influx, indicating that glymphatic transport may be supported by multiple complementary mechanisms [14, 16].

Finally, an important translational challenge is the determination of the extent to which the mechanistic insights derived from rodent models apply to human neurological disease. Recent human imaging studies have provided evidence consistent with several key features of glymphatic transport, including its perivascular organization and modulation by sleep, but currently available imaging approaches capture different aspects of perivascular fluid dynamics rather than directly measuring glymphatic exchange or waste clearance [31]. Furthermore, although multiple CSF efflux pathways have now been identified, their quantitative contributions to overall brain clearance and their functional integration remain poorly defined. A comprehensive three-dimensional map of the human glymphatic–lymphatic network is still lacking, and whether regional differences in clearance efficiency contribute to selective vulnerability in neurological diseases remains unknown.

Despite these outstanding questions, several fundamental concepts have gained broad acceptance. Brain fluid and solute transport is dynamically regulated by physiological states such as sleep, aging, and vascular function, and disruption of these processes is increasingly associated with neurological disorders. Future advances in quantitative imaging, computational modeling, and standardized experimental paradigms will be essential for refining the glymphatic–lymphatic framework and facilitating its translation into human physiology and neurological disease.

### Physiological regulation of the glymphatic–lymphatic axis

Rather than functioning as a passive drainage system, the efficiency of this clearance network is continuously shaped by multiple physiological and pathological regulators. Among these, sleep represents one of the most robust physiological modulators of glymphatic transport, while mechanical forces, systemic physiological states, and pathological conditions regulate different components of glymphatic and lymphatic function. Together, these factors determine the efficiency of brain fluid transport, waste clearance, and CNS immune surveillance [37, 38].

#### Sleep, circadian rhythms, and glymphatic function

Sleep is widely regarded as one of the most important modulators of glymphatic function and has emerged as a major link between brain clearance and neurodegeneration. Sleep architecture critically influences glymphatic transport, with deep non-rapid eye movement sleep representing the physiological state most strongly associated with enhanced CSF influx into the brain parenchyma and increased clearance of interstitial solutes [39, 40]. Reduced average noradrenergic tone during non-rapid eye movement sleep may favor expansion of the interstitial space, while infraslow norepinephrine oscillations drive slow cerebral vasomotion and coordinated changes in cerebral blood volume and CSF dynamics, thereby promoting glymphatic transport in mice [40, 41].

**Fig. 2:**
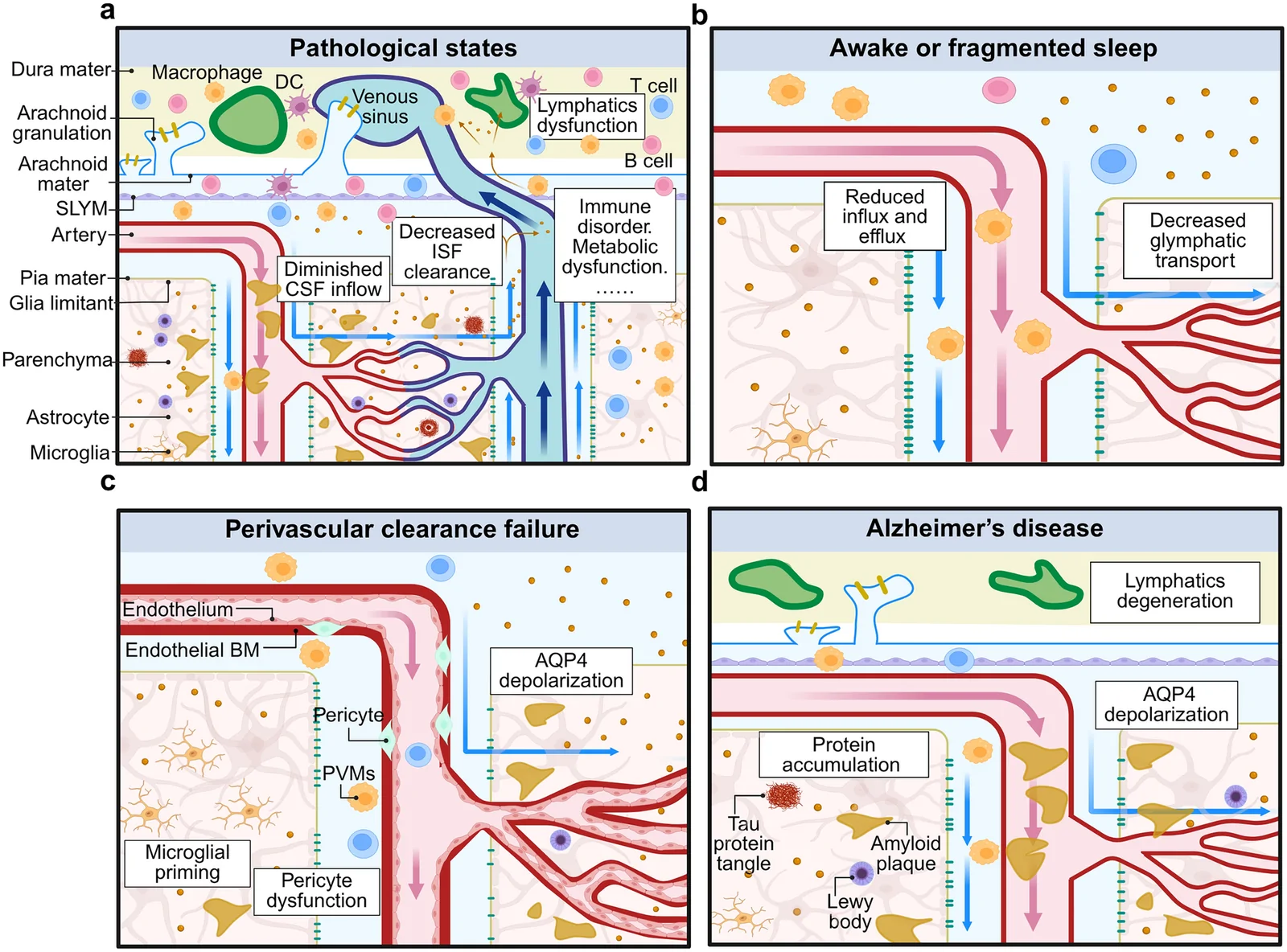
Structure, immune surveillance, and functional dynamics of the glymphatic–lymphatic axis in different conditions. **a** Schematic overview of disease-associated alterations in CSF–ISF exchange, solute transport, and immune cell trafficking at the brain-meningeal interface. **b** Sleep-dependent regulation of glymphatic transport. Sustained arousal or sleep disruption is associated with reduced glymphatic-related transport through mechanisms involving noradrenergic tone, vascular dynamics, interstitial-space regulation, and potentially AQP4 polarization. **c** Perivascular clearance failure across neurological disorders. Diverse pathological insults perturb CSF dynamics, leading to abnormal CSF influx or efflux, enlargement of perivascular spaces, and impaired molecular clearance. **d** Glymphatic and meningeal lymphatic dysfunction in Alzheimer’s disease. Amyloid accumulation, astroglial dysfunction, and structural and functional impairment of meningeal lymphatic vessels converge to disrupt perivascular clearance and alter immune surveillance at the brain–meningeal interface. SLYM, subarachnoid lymphatic-like membrane; AQP4, aquaporin-4; CSF, cerebrospinal fluid; BM, basement membrane; PVMs, perivascular macrophages. (Created with BioRender.com)

Increasing evidence suggests that the glymphatic activity is also subject to circadian regulation. Circadian oscillations may influence AQP4 polarization and the efficiency of perivascular transport, suggesting that glymphatic transport and waste clearance may vary across the circadian cycle [42]. Accordingly, disruption of normal sleep architecture, circadian misalignment, and age-related reductions in slow-wave sleep have emerged as important contributors to impaired brain clearance [40] **(**Fig. 2b**)**.

Sleep disturbance has therefore been recognized not only as a consequence of neurodegeneration but also as a potential contributor to disease initiation and progression. Accumulating evidence supports a bidirectional relationship between sleep disturbance and neurodegenerative diseases such as AD [31, 40]. Chronic sleep disruption may impair the clearance of pathological proteins, including Aβ and tau, whereas accumulation of these proteins may further perturb sleep regulation, establishing a self-reinforcing cycle that could accelerate disease progression [43]. Collectively, these findings position sleep as a key physiological regulator of the glymphatic–lymphatic axis and a clinically relevant, potentially modifiable factor linking lifestyle, aging, and neurodegeneration.

#### Mechanical and systemic determinants of glymphatic–lymphatic transport

In addition to sleep, multiple mechanical and systemic factors contribute to the regulation of glymphatic transport. In mice, 40-Hz sensory stimulation has been shown to enhance glymphatic transport through mechanisms involving adenosine signaling, vascular dynamics, and meningeal lymphatic drainage [44, 45]. Consistent with these findings, experimental attenuation of cerebral arterial pulsation reduces paravascular CSF–ISF exchange in mice, supporting arterial pulsatility as an important, yet not exclusive, mechanical contributor to periarterial CSF transport [46]. Parenchymal border macrophages, including perivascular and leptomeningeal macrophages, provide an additional regulatory layer by maintaining the perivascular extracellular matrix and arterial motion, thereby supporting CSF flow dynamics [47].

Other physiological factors, such as body posture [48], as well as external factors including anesthetic agents [49], can influence glymphatic tracer transport. Although the specific regulatory mechanisms remain incompletely understood, potential mechanisms may include astrocytic AQP4 polarization, neurovascular coupling, and dynamic regulation of vascular tone.

#### Pathophysiological modifiers of the glymphatic–lymphatic axis

Beyond physiological regulation, pathological processes can also profoundly influence glymphatic transport. Traumatic brain injury provides a well-characterized example in which persistent neuroinflammation and neurovascular dysfunction are accompanied by reactive astrogliosis and loss of perivascular AQP4 polarization [50–53]. These alterations have been associated with reduced perivascular CSF influx and impaired glymphatic transport, thereby compromising the clearance of interstitial solutes from the brain [51]. More broadly, vascular dysfunction may impair brain fluid and solute transport by reducing the arterial wall pulsatility that contributes to periarterial CSF influx and by disrupting neurovascular unit integrity [54]. Importantly, AQP4 is dynamically regulated during neuroinflammation. As highlighted by Fukuda and Badaut, changes in AQP4 expression and localization vary according to the disease model and stage of injury, reflecting its distinct roles during edema formation and resolution [52].

This pathological interplay is central to age-related decline in brain clearance. Aging represents a major modifier of the glymphatic–lymphatic axis, characterized by a triad of detrimental changes: reduced vascular compliance, loss of astrocytic AQP4 polarization, and structural and functional impairment of mLVs, which collectively contribute to a progressive decline in the clearance of interstitial solutes and metabolic waste from the brain [33, 54–56]. In mice, aging is associated with impaired glymphatic function, characterized by reduced paravascular CSF influx and diminished interstitial solute clearance, accompanied by loss of perivascular AQP4 polarization and increased reactive astrogliosis [54]. However, the causal contributions of these age-related changes to glymphatic dysfunction remain incompletely understood. In humans, MRI studies using diffusion tensor image analysis along the perivascular space (DTI-ALPS) have generally reported lower ALPS indices in older adults, suggesting age-related alterations in perivascular fluid transport [57, 58]. However, because DTI-ALPS provides an indirect measure of water diffusivity along the perivascular space rather than fluid flow itself, it cannot determine the directionality of perivascular transport or directly quantify glymphatic function [59].

In addition to chronic aging-related changes, acute neurological injuries also provide important evidence of glymphatic dysfunction and its pathological consequences. In a rodent model of traumatic brain injury, Iliff et al*.* showed that the glymphatic pathway function was reduced by approximately 60% and remained impaired for at least one month, suggesting long-term disruption of glymphatic clearance pathways [50]. Consistent with these findings, Bolte et al*.* demonstrated that mild traumatic brain injury rapidly impaired meningeal lymphatic drainage, that increased intracranial pressure contributed to this dysfunction, and that restoration of meningeal lymphatic function in aged mice ameliorated injury-induced gliosis [60]. Experimental studies further suggest that pre-existing or developmental meningeal lymphatic dysfunction can worsen neuroinflammation, glial activation, and behavioral outcomes following traumatic brain injury [60, 61].

Biological sex may modify glymphatic-related brain fluid dynamics through effects on sleep, vascular and astrocytic physiology, and hormonal signaling. Human MRI studies have generally reported lower DTI-ALPS indices in males than in females, although head size and white-matter microstructure may partly explain this association [58, 62, 63]. By contrast, direct tracer experiments in healthy mice detected no significant sex difference in glymphatic influx [64], suggesting that the observed sex effects may depend on the imaging marker, population, and disease context. Whether sex-dependent regulation of the glymphatic–lymphatic axis contributes to the higher prevalence of AD in females [65] and PD in males [66] remains unknown.

Despite these advances, several important mechanistic and translational questions remain unresolved, including whether age-associated glymphatic decline represents an unavoidable biological trajectory or a modifiable process amenable to intervention, and whether clearance impairment associated with aging, brain injury, or neurodegenerative disease can be restored or reversed. More fundamentally, these questions cannot be fully addressed by viewing brain clearance as an isolated intracranial process.

## Functional integration of the glymphatic–lymphatic axis with peripheral immunity and metabolism

Increasing evidence suggests that the glymphatic–lymphatic axis functions not only as a brain clearance pathway, but also as a broader physiological network linking the CNS with peripheral immune and metabolic systems. Through anatomically organized drainage pathways and reciprocal molecular signaling, this system contributes to immune surveillance, metabolic regulation, and neurovascular homeostasis under both physiological and pathological conditions. In the following sections, we discuss how CNS-derived signals shape peripheral responses and, conversely, how peripheral physiological states regulate glymphatic function and brain homeostasis.

### CNS-to-periphery signaling

The CNS was historically regarded as an immune-privileged compartment. However, accumulating evidence has revised this classical view by revealing extensive immunological communication between the CNS and peripheral compartments [67]. Such communication occurs through specialized immune interfaces at the meninges, dural sinuses, blood–brain barrier, choroid plexus, and skull bone marrow, which collectively contribute to CNS immune surveillance and homeostasis. Beyond parenchymal microglia, diverse immune populations, including dendritic cells, T cells, B cells, and natural killer cells, have been identified predominantly within these CNS border compartments [68, 69]. By contrast, immune-cell access to the brain parenchyma is tightly restricted under physiological conditions, although infiltration can increase during neuroinflammation and neurodegenerative disease [68–70]. The abundance, cellular origin, and functional significance of these infiltrating populations, particularly during chronic neurodegeneration, remain incompletely defined [71, 72].

As the peripheral immune compartment closest to the CNS, the skull bone marrow serves as one of the earliest recipients of brain-derived signals. It receives CSF-borne molecules and other CNS-derived signals through dura–skull channels, thereby modulating local hematopoiesis and immune responses [73, 74]. In mouse models of bacterial meningitis, pathogens present in the CSF can enter the skull bone marrow and trigger local hematopoietic activation [20]. Likewise, pronounced immune cell accumulation has been observed in skull bone marrow regions adjacent to glioblastomas [75], further supporting dynamic communication between intracranial pathology and local bone marrow immunity.

CNS-derived molecules reaching the CSF and meningeal compartments can be transported through meningeal lymphatic vessels to the deep cervical lymph nodes, which serve as key peripheral immune sites for CNS immune surveillance [76]. During CNS inflammatory diseases, including bacterial meningitis, drainage of CNS-derived antigens and inflammatory mediators to the cervical lymph nodes can amplify both peripheral and central immune responses, thereby contributing to disease progression [12]. In experimental models of traumatic brain injury and ischemic stroke, disruption of brain-to-cervical lymph-node signaling or surgical removal of cervical lymph nodes reduced specific inflammatory responses and tissue injury [77, 78]. Similarly, in the A53T mouse model of PD, CNS-derived oligomeric α-synuclein drains through mLVs to the deep cervical lymph nodes, where it activates macrophages and is associated with lymph node enlargement and macrophage accumulation [76]. Together, these findings indicate that lymphatic drainage not only removes CNS-derived molecules, but can also shape peripheral immune responses.

Beyond these dedicated drainage pathways, alterations in CNS immune status can also induce immune changes in peripheral organs [79]. In experimental models, chronic stress can lead to splenomegaly and induce monocytosis originating from the spleen and bone marrow [80]. Consistent with this neuroimmune connection, viral transneuronal tracing has identified brain regions with polysynaptic connections to the spleen, including the paraventricular nucleus of the hypothalamus and the central nucleus of the amygdala [81]. In addition, experimental studies in mice have shown that stress- and immune-responsive brain circuits regulate gut microbial composition and intestinal immune homeostasis through autonomic pathways [82, 83]. Whether the brain-gut axis or other brain-organ axes communicate via the lymphatic system remains to be fully elucidated.

Collectively, these findings indicate that the CNS is not immunologically isolated but can export antigens, immune mediators, and pathological proteins through anatomically organized drainage pathways, thereby shaping the peripheral immune surveillance and responses [12, 73, 76]. The glymphatic–lymphatic axis therefore functions not only as a clearance system, but also as an important communication route through which the CNS conveys signals to and modulates peripheral immunity.

### Periphery-to-CNS modulation

Emerging evidence indicates that the glymphatic–lymphatic axis is not only an output pathway through which the CNS communicates with the periphery, but is also modulated by peripheral physiological and pathological signals that influence brain homeostasis. Immune cell trafficking, microbial and metabolic signaling, and circulating extracellular vesicles collectively provide multiple routes by which peripheral states may regulate glymphatic transport, neurovascular function, and CNS immunity.

The skull bone marrow–meningeal interface provides a direct anatomical route linking peripheral hematopoietic activity to CNS border immunity. During CNS injury and inflammation, skull bone marrow-derived myeloid cells can migrate through microscopic vascular channels into the dura and other meningeal compartments [84]. In mouse models of multiple sclerosis and stroke, skull and vertebral bone marrow supply monocytes and neutrophils to the meninges and, under selected pathological conditions, the CNS parenchyma; these cells exhibit a less proinflammatory phenotype than their counterparts from distant hematopoietic compartments [85]. In parallel, ACE points provide distinct openings through which dural immune cells can cross the arachnoid barrier and enter the subarachnoid space during neuroinflammation [19]. This interface also supports adaptive immune homeostasis, as dural B cells can arise locally from the skull bone marrow and may contribute to the recognition of brain-derived antigens and immune tolerance [86]. In cancer, skull-associated routes appear to have context-dependent effects. Intracranial glioblastomas and adjacent skull bone marrow share T-cell clones, while local mobilization of skull bone marrow-derived immune cells with the CXCR4 antagonist AMD3100 enhances antitumor responses in experimental models [87]. Conversely, skull osseous channels have been implicated as potential routes through which leukemia and breast cancer cells may reach the meninges and promote CNS dissemination [88, 89].

In addition to serving as drainage sites for CNS-derived antigens, cervical lymph nodes support the activation and differentiation of immune cell populations capable of regulating CNS inflammation. For example, IL-10-dependent Tr1 cells induced in cervical lymph nodes have been shown to suppress astrocyte activation and alleviate chronic CNS inflammation in mouse models, including a model of progressive multiple sclerosis [90].

Beyond immune regulation, peripheral organs may influence glymphatic function through microbial and metabolic signaling. Gut microbiota and their metabolites can influence blood–brain barrier integrity and microglial homeostasis, thereby providing potential routes through which peripheral microbial states affect CNS immunity [91, 92]. Microbiota-derived short-chain fatty acids can further influence microglial maturation, peripheral circadian clocks, and sleep in animal models [92–94]. These effects may, in turn, indirectly modulate physiological states relevant to glymphatic transport, although direct evidence remains limited. In addition, microbiota-derived bacterial extracellular vesicles have emerged as important mediators of gut-brain communication [95]. By transporting microbial lipids, proteins, nucleic acids, and other bioactive cargo across biological barriers, microbiota-derived bacterial extracellular vesicles can influence blood–brain barrier integrity and glial function, thereby altering neuroimmune signaling and neurovascular unit homeostasis [95, 96]. These effects may, in turn, indirectly influence CSF–ISF exchange and glymphatic clearance.

Systemic metabolic and cardiovascular disorders have emerged as important disruptors of glymphatic clearance. Accumulating evidence suggests that diabetes and obesity can be associated with altered glymphatic transport [97–99], although the direction and magnitude of these changes may depend on the model, disease duration, and brain region. In diabetic rat models, brain tracer clearance became progressively impaired with increasing disease duration [100]. In a separate study, type 2 diabetes delayed the clearance of CSF-delivered tracers from the brain interstitium, particularly in the hippocampus and hypothalamus, and greater tracer retention was associated with more severe cognitive deficits [101]. Cardiovascular dysfunction may likewise impair glymphatic transport through alterations in vascular hemodynamics [102]. Increased arterial stiffness and reduced vascular compliance may reduce pulsatile vascular wall motion, which is considered one of several important contributors to periarterial CSF transport [103].

Beyond these established associations, circulating extracellular vesicles represent an additional potential route of periphery-to-CNS communication. In mice, systemically administered extracellular vesicles can cross the intact blood–brain barrier, although their rates of brain entry vary according to vesicle origin and can be altered by systemic inflammation [104]. Complementary genetic reporter studies have shown that hematopoietic cell-derived extracellular vesicles can transfer functional RNA to neurons, an event that is rare under physiological conditions but markedly enhanced during peripheral inflammation [105]. These findings support extracellular vesicle-mediated communication between the periphery and the brain, although whether circulating extracellular vesicles directly regulate glymphatic transport remains unknown.

Despite growing insights into brain-periphery communication, important questions remain regarding the directionality, hierarchy, and causal significance of these interactions. Whether systemic dysfunction drives glymphatic impairment or primary clearance failure disrupts peripheral homeostasis remains incompletely understood. Resolving these questions is particularly important because disruption of this integrated network is increasingly implicated in the pathophysiology of neurodegenerative diseases.

## Glymphatic–lymphatic dysfunction in neurodegeneration

Disruption of the glymphatic–lymphatic axis has emerged as a common feature of many neurodegenerative diseases. Increasing evidence from both experimental models and human studies indicates that impaired glymphatic–lymphatic function may compromise the clearance of pathogenic proteins, disrupt neurovascular and immune homeostasis, and contribute to disease progression [6] (Fig. 2a). Aging and neurodegenerative diseases are associated with progressive structural and functional alterations of the glymphatic–lymphatic axis that may ultimately impair the clearance of interstitial solutes and pathogenic proteins from the brain [54, 55].

Human arachnoid granulations exhibit age-related changes in number, size, and distribution. In PD, increased arachnoid-granulation burden has been associated with motor impairment, cognitive dysfunction, and sleep disturbance [106]. In addition, impaired peri-olfactory CSF clearance has been associated with sleep disturbance and cognitive dysfunction and may partly mediate the relationship between them [107].

Vascular dysfunction is another important factor contributing to glymphatic–lymphatic impairment during aging and neurodegenerative diseases. Accumulating evidence indicates that neurovascular dysfunction can arise before the clinical onset of neurodegenerative disease, particularly in AD and related cognitive disorders **(**Fig. 2c**)** [108, 109]. During the preclinical or early stages of AD, the neurovascular unit may already exhibit pericyte injury and blood–brain barrier dysfunction, as reflected by elevated CSF levels of soluble PDGFRβ and increased hippocampal blood–brain barrier permeability [108]. As the disease progresses, additional neurovascular alterations may emerge, including reduced pericyte coverage [110], extravascular deposition of plasma proteins such as fibrin [110], loss of endothelial tight-junction proteins [111], reduced perivascular AQP4 localization [56], and microglial activation [112]. Collectively, these changes may reflect an early compromise in perivascular clearance and local immune homeostasis, even in the absence of overt neuronal loss. As disease progresses and neurological symptoms emerge, neurovascular unit disruption may intensify. Significant pericyte loss and sustained blood–brain barrier hyperpermeability can lead to persistent extravasation of blood-derived components. Concurrent glymphatic and meningeal lymphatic dysfunction may contribute to the accumulation of pathogenic proteins [113]; however, the relative contribution of each pathway remains incompletely defined.

The glymphatic and meningeal lymphatic pathways have been implicated in the transport and clearance of several neurodegeneration-associated proteins [6]. Experimental tracer studies have demonstrated that soluble interstitial Aβ and extracellular tau can be cleared through glymphatic or paravascular pathways [1, 50]. With aging, mLV drainage declines [55], while experimental disruption of meningeal lymphatic function in 5×FAD mice exacerbates Aβ-associated pathology and alters microglial responses [114]. In A53T mice, blockade of meningeal lymphatic drainage similarly aggravates glymphatic dysfunction and perivascular α-synuclein accumulation, although direct lymphatic clearance of α-synuclein was not quantified [113]. Conversely, induction of dural lymphangiogenesis enhances soluble Aβ clearance in a transgenic mouse model of AD [115] **(**Fig. 2d**)**. Early glymphatic dysfunction has also been detected in a TDP-43 mouse model of amyotrophic lateral sclerosis, although whether this dysfunction directly contributes to TDP-43 accumulation remains unresolved [116]. Beyond these fluid-clearance pathways, the skull bone marrow undergoes substantial age-related remodeling. Recent work indicates lifelong expansion of the skull bone marrow accompanied by vascular growth, although the implications of these changes for CNS–skull bone marrow communication remain unclear [117].

Neurodegenerative pathology also exhibits pronounced spatial selectivity. Aβ plaques typically appear first in neocortical regions [118], while tau and α-synuclein inclusions follow distinct topographical patterns that depend on disease context [119, 120]. One possible contributor to this spatial vulnerability is regional heterogeneity in glymphatic–lymphatic clearance capacity, which may interact with local protein production and aggregation to promote protein retention in susceptible brain regions. Such regional differences in clearance could potentially arise from variations in vascular architecture, perivascular-space organization, astrocytic AQP4 polarization, and anatomical proximity to downstream CSF outflow pathways [121]. Indirect support for this hypothesis comes from emerging human and experimental evidence demonstrating region-specific abnormalities in tracer transport and pathological protein accumulation. Following intrathecal gadobutrol administration, patients with idiopathic normal pressure hydrocephalus exhibited greater tracer retention than reference subjects at 24 h in the cerebral white matter, basal ganglia, corpus callosum, limbic structures, and hippocampus, consistent with regionally delayed tracer clearance [122]. In A53T mice, reduced CSF tracer influx is accompanied by perivascular α-synuclein accumulation in the substantia nigra and loss of perivascular AQP4 polarization [113]. However, these observations do not establish whether regional clearance impairment precedes and promotes pathological protein deposition or instead develops secondary to local protein accumulation and neurovascular dysfunction. Regional variation in clearance capacity may therefore contribute to, rather than independently determine, the spatial vulnerability of neurodegenerative pathology.

Overall, these findings support a model in which neurodegenerative disease is shaped not only by neuron-intrinsic defects but also by progressive failure of neurovascular and clearance homeostasis. Impaired removal of pathogenic proteins, together with immune dysregulation, may establish a self-reinforcing cycle that amplifies regional vulnerability and accelerates disease progression. These observations naturally raise an important translational question: can restoration of glymphatic–lymphatic function interrupt this pathogenic cycle and modify disease progression?

## Therapeutic targeting and biomarkers of the glymphatic–lymphatic axis

### Therapeutic targeting of the glymphatic–lymphatic axis

**Fig. 3:**
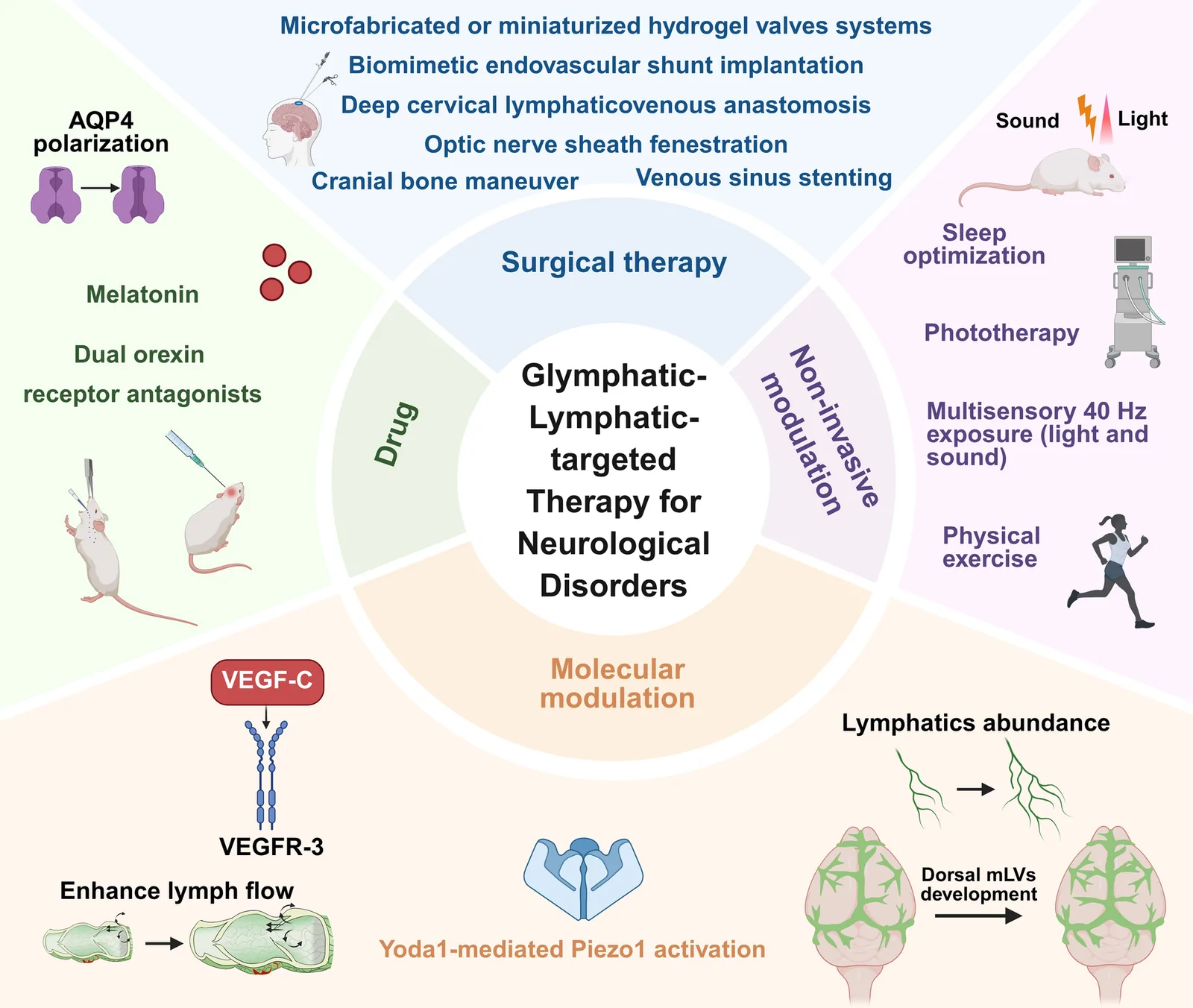
Therapeutic strategies targeting the glymphatic–lymphatic axis in neurological disorders. Pharmacological, surgical, genetic, and non-invasive interventions target multiple components of the glymphatic–lymphatic axis to restore CSF and ISF dynamics, improve molecular clearance, and promote neurovascular and immune homeostasis. (Created with BioRender.com)

The proposal that glymphatic–lymphatic dysfunction may contribute to neurodegeneration has prompted growing interest in therapeutic strategies aimed at restoring brain clearance. Although many mechanistic questions remain unresolved, accumulating evidence suggests that multiple components of the glymphatic–lymphatic axis may be therapeutically modifiable [123, 124]. These approaches span molecular, pharmacological, device-based, and surgical interventions but differ considerably in their stages of development and strength of supporting evidence. The major therapeutic approaches are summarized in Fig. 3 and Table 1.

**Table 1:** Potential interventions to modulate the glymphatic–lymphatic system in neurological disorders

Interventions	Strategy	Primary targets	Proposed mechanism	Current evidence	Key limitations	References
Drugs	Melatonin	Sleep–circadian regulation and glymphatic transport	Restores circadian AQP4 polarization and enhances glymphatic tracer clearance	From animal model studies	Lack of definitive evidence for clinical efficacy	[125, 126]
	Agomelatine	Sleep–circadian regulation and AQP4 polarization	Promotes restoration of perivascular AQP4 polarization	From an animal and retrospective human study	Clinical efficacy requires prospective validation	[127]
	Dual orexin receptor antagonists	Orexin signaling, sleep regulation, and protein homeostasis	Improves sleep and modulates Aβ and tau-related biomarkers or pathology	From animal and acute human biomarker studies	Long-term disease-modifying efficacy is unknown	[128, 129]
Non-invasive modulation	Sleep optimization	Glymphatic-related brain fluid dynamics	Modulates interstitial-space, vascular, and CSF dynamics	From animal and human studies	Effects on net molecular clearance and disease modification remain uncertain	[40, 130, 131]
	40-Hz stimulation	Glymphatic–lymphatic axis	Enhances CSF influx and interstitial solute efflux	From an animal study	Mechanisms incompletely understood	[45]
	Near-infrared phototherapy	mLVs	Promotes lymphatic clearance	From an animal study	Lack of human studies	[132]
Molecular modulation	VEGF-C	mLVs	Induces meningeal lymphangiogenesis	From animal studies	Delivery, safety, and context- or timing-dependent effects	[55, 114, 133, 134]
	Piezo1	mLVs	Enhances CSF outflow and meningeal lymphatic transport through Piezo1-mediated mechanotransduction	From an animal study	Human efficacy, safety, and target selectivity remain unknown	[135]
Surgical therapy	Cranial bone maneuver	mLVs	Promotes meningeal lymphangiogenesis and lymphatic drainage	From animal studies	Highly invasive; safety, feasibility, and efficacy in humans remain unknown	[136, 137]
	Biomimetic CSF shunts	CSF outflow pathways	Provides pressure-responsive, one-way CSF diversion through engineered shunt systems	Engineering/preclinical and early human studies	Long-term safety, durability, and comparative efficacy remain uncertain	[138–140]
	Venous sinus stenting	Dural venous outflow	Reduces venous pressure	From a human study	Effect on glymphatic clearance unknown	[141]
	Optic nerve sheath fenestration	Perioptic CSF pressure/local CSF compartment	Local decompression	From human studies	Local rather than whole-brain effect	[142, 143]
	Deep cervical lymphaticovenous anastomosis	Downstream drainage	Improves lymphatic outflow	From early studies in humans	Long-term efficacy unknown	[144, 145]

Physiological and lifestyle factors, including sleep, exercise, diet, and circadian rhythms, represent readily accessible modulators of the glymphatic–lymphatic axis. In mice, sleep has been reported to expand the interstitial space by approximately 60%, accelerating the clearance of Aβ and other metabolites [40]. In humans, sleep and sleep deprivation have been associated with changes in brain fluid dynamics and tracer kinetics, including overnight brain-to-plasma transport of Aβ and tau [130] and delayed intrathecal tracer clearance after one night of total sleep deprivation [131]. One proposed mechanism involves sleep- and circadian-dependent changes in AQP4 polarization. Hablitz et al*.* reported that perivascular AQP4 polarization peaked during sleep [42], whereas Frey et al., using quantitative immunogold electron microscopy, found no significant diurnal variation in AQP4 localization, implicating additional or alternative mechanisms [146]. Improving sleep and treating sleep disorders may support glymphatic-related brain fluid dynamics, although current human evidence is derived largely from observational and longitudinal imaging studies [39, 147, 148]. Other physiological interventions, including voluntary exercise [149] and omega-3 polyunsaturated fatty acid supplementation [150], have been shown in aged or experimentally Aβ-challenged mice to enhance glymphatic transport and facilitate brain Aβ clearance, accompanied by increased or preserved perivascular AQP4 polarization and reduced glial activation.

Pharmacological modulation of sleep and circadian rhythms provides a complementary approach to physiological interventions. Melatonin has been shown to restore circadian rhythms, regulate AQP4 polarization, and enhance glymphatic clearance in animal models [125, 126], although direct evidence that melatonin enhances glymphatic clearance in humans is currently lacking. More recently, agomelatine, a melatonergic antidepressant with sleep- and circadian-modulating properties, was associated with a higher DTI-ALPS index and improved motor performance in a retrospective study of patients with PD, while complementary experiments in MPTP-treated mice linked these effects to BMAL1-dependent restoration of AQP4 polarization [127]. The dual orexin receptor antagonist suvorexant has been shown to acutely reduce CSF Aβ levels and tau phosphorylation in cognitively unimpaired adults [128], while dual orexin receptor antagonism attenuates tau pathology in mouse models [129]. In patients with insomnia disorder, long-term treatment with daridorexant was well tolerated and produced sustained improvements in sleep onset, sleep maintenance, and daytime functioning for up to 52 weeks, although this study did not assess glymphatic transport or neurodegenerative biomarkers [151].

Although the disease-modifying effects of these strategies in neurodegenerative disorders remain uncertain, interventions that directly regulate CSF outflow and intracranial fluid dynamics have shown clear clinical utility in selected CSF circulation disorders. CSF shunting is an established surgical treatment for appropriately selected patients with idiopathic normal pressure hydrocephalus. Among the available procedures, ventriculoperitoneal shunting is commonly performed worldwide and diverts ventricular CSF into the peritoneal cavity for absorption, thereby improving CSF drainage and intracranial fluid dynamics [152]. Building on the principle of CSF diversion, several biomimetic shunt devices have been developed to more closely reproduce pressure-dependent physiological CSF outflow. Microfabricated shunts use engineered microvalve and microneedle arrays to enable pressure-dependent, one-way CSF drainage, thereby mimicking the function of arachnoid villi and granulations [138]. Hydrogel valve systems use deformable hydrogel diaphragms to achieve threshold-dependent opening and reduce reverse flow [139]. Transdural endovascular shunts provide a minimally invasive strategy to divert CSF from a subarachnoid cistern into the venous system, thereby avoiding conventional ventriculoperitoneal drainage [140]. Microfabricated and hydrogel valve systems remain largely at the engineering and preclinical stages, whereas transdural endovascular shunts have entered early clinical investigation for communicating hydrocephalus. However, their long-term safety, durability, and comparative efficacy require further validation.

Several biomimetic approaches discussed above are designed to facilitate CSF movement from the SAS into dural venous outflow pathways, whereas conventional shunts redirect CSF to extracranial absorption sites. Once CSF reaches these drainage interfaces, its onward clearance depends on venous and lymphatic pathways that ultimately return fluid and solutes to the peripheral circulation. Accordingly, therapeutic strategies targeting dural venous and meningeal lymphatic outflow have attracted increasing attention. Intracranial venous sinus stenting, which restores venous outflow and reduces venous sinus pressure gradients, has become a treatment option for selected patients with idiopathic intracranial hypertension [141], particularly when venous sinus stenosis contributes to impaired CSF absorption and elevated intracranial pressure. However, current clinical evidence is derived predominantly from observational studies and remains limited by heterogeneous patient selection and insufficient long-term comparative data. Furthermore, direct evidence demonstrating improved glymphatic clearance after venous sinus stenting is still lacking.

mLVs represent another potential target for improving dural-to-peripheral CSF and solute drainage. In mouse models, 40-Hz multisensory stimulation enhanced cortical CSF influx and interstitial solute efflux, accompanied by mLV dilation and increased accumulation of brain-derived amyloid in cervical lymph nodes [45], whereas near-infrared phototherapy enhanced mLV drainage and improved lymphatic endothelial function [132]. An experimental cranial bone maneuver involving controlled displacement of a surgically created cranial bone flap promoted mLV lymphangiogenesis and drainage in 5×FAD mice and, through an mLV-dependent mechanism, reduced cerebral Aβ deposition and neuroinflammation and improved memory performance [136]. In parallel, molecular strategies have focused on promoting meningeal lymphatic development and function. Acting through VEGFR-3 on lymphatic endothelial cells, vascular endothelial growth factor-C (VEGF-C) is a key regulator of mLV development, maintenance, and plasticity [133]. In aged mice, VEGF-C treatment enhanced the drainage of CSF macromolecules to the deep cervical lymph nodes and improved cognitive performance [55]. In 5×FAD mice, VEGF-C-mediated expansion of mLVs enhanced the efficacy of anti-Aβ immunotherapy [114]. The effects of VEGF-C are nevertheless context- and timing-dependent: in a mouse model of ischemic stroke, sustained VEGF-C expression before injury enhanced lymphatic drainage and improved neurological outcomes, whereas acute administration after stroke did not produce comparable benefits [134]. More recently, the mechanosensitive ion channel Piezo1 has been implicated in mLV regulation, with Piezo1 activation enhancing CSF outflow and lymphatic transport and reducing intracranial pressure in mice [135]. Despite these encouraging findings, nearly all evidence supporting direct enhancement of mLV function is derived from rodents, and the safety and therapeutic efficacy of these approaches in humans remain unknown.

Beyond dural venous and meningeal lymphatic routes, CSF may also drain through other extracranial lymphatic or perineural pathways, including cribriform plate-associated nasal lymphatics, optic nerve sheath-associated routes, and deep cervical lymphatic drainage. Optic nerve sheath fenestration improves visual outcomes in patients with idiopathic intracranial hypertension by locally decompressing the perioptic CSF space [142, 143]. However, whether such local decompression can simultaneously promote CSF outflow to peripheral tissues remains unclear. More peripheral approaches have also been proposed to enhance CSF outflow. Deep cervical lymphaticovenous anastomosis has been proposed as a microsurgical strategy to facilitate downstream lymphatic outflow by connecting deep cervical lymphatic vessels to adjacent veins [144]. Although a small, uncontrolled prospective cohort reported short-term cognitive changes after surgery [145], its limited sample size, short follow-up, and lack of randomization and a concurrent control group preclude conclusions regarding efficacy.

Overall, the current therapeutic landscape remains heterogeneous. While interventions such as sleep optimization are supported by both experimental and clinical evidence, most strategies directly targeting glymphatic or meningeal lymphatic pathways, including VEGF-C, Piezo1 activation, and phototherapy, remain at the preclinical proof-of-concept stage. A limited number of surgical approaches have entered early clinical evaluation, although their effects on glymphatic–lymphatic transport remain uncertain. Nevertheless, these studies provide preclinical proof-of-concept that selected components of the glymphatic–lymphatic axis can be experimentally modulated, supporting the possibility that modulation of endogenous brain clearance pathways may complement existing protein-targeted therapies. As these therapeutic strategies move toward clinical translation, reliable biomarkers capable of assessing glymphatic–lymphatic function and monitoring treatment responses will become increasingly important.

### Biomarkers of the glymphatic–lymphatic axis

Several imaging and physiological biomarkers have been proposed to assess the glymphatic–lymphatic functions in humans. The DTI-ALPS index is an indirect diffusion-derived metric initially proposed to reflect water diffusivity along perivascular spaces [153]. However, its biological interpretation remains uncertain because it is influenced by white-matter fiber geometry and other microstructural features. In addition, a recent comparison with intrathecal contrast-enhanced MRI found only limited correspondence between the ALPS index and tracer-based measures of CSF enrichment and clearance [154, 155]. CSF tracer-based kinetic imaging offers a more direct assessment of solute distribution and clearance dynamics but remains invasive and is currently limited to selected clinical and research settings [156]. In addition, simultaneous EEG and functional MRI studies have demonstrated that slow-wave activity during non-rapid eye movement sleep is temporally coupled to hemodynamic and CSF signal oscillations, providing an indirect physiological correlate of sleep-dependent brain fluid dynamics [157, 158]. Collectively, these biomarkers provide complementary information regarding different aspects of glymphatic physiology; however, none currently enables direct, quantitative assessment of glymphatic transport or waste clearance in humans.

Developing robust and clinically accessible biomarkers of CNS clearance remains a major priority. Such biomarkers will be essential not only for identifying individuals most likely to benefit from clearance-targeted interventions, monitoring longitudinal treatment responses, and defining therapeutic windows across disease stages, but also for accelerating the clinical translation of glymphatic–lymphatic therapies. Together, advances in therapeutic targeting and biomarker development are beginning to transform the glymphatic–lymphatic axis from a mechanistic concept into an emerging translational framework. However, realizing this translational potential will require closer integration of mechanistic discovery, quantitative biomarkers, and well-designed clinical studies. These priorities define the next major challenges for the field.

## Future directions and conceptual synthesis

Despite substantial progress, major questions remain regarding the organization, the regulation, and the physiological significance of the glymphatic–lymphatic axis. First, important mechanistic questions remain unresolved. Future studies should define the quantitative contributions of individual clearance pathways, establish causal relationships between impaired clearance and disease progression, and determine how these pathways differ across species and neurological disorders.

Second, progress in the field will depend on the development of reliable methods for assessing glymphatic–lymphatic functions in humans. Standardized imaging protocols and quantitative human biomarkers capable of measuring clearance function in vivo will be essential for comparing results across studies, monitoring therapeutic responses, and facilitating clinical translation.

Finally, future therapeutic development will require integration of mechanistic insights with translational research. More broadly, the glymphatic–lymphatic axis provides a systems-level framework linking waste clearance, vascular biology, immune surveillance, and brain-periphery communication. Rather than replacing existing disease-modifying therapies, future strategies aimed at restoring endogenous clearance pathways may complement protein-targeted interventions by improving overall neurovascular and clearance homeostasis. Continued integration of mechanistic studies, translational biomarkers, and clinical trials will determine whether modulation of the glymphatic–lymphatic axis can ultimately improve outcomes in neurodegenerative diseases.

## Conclusions

Over the past decade, the glymphatic–lymphatic axis has evolved from a largely hypothetical model into an experimentally supported framework for understanding brain fluid transport, solute clearance, and communication between intracranial and extracranial compartments. Accumulating evidence from experimental models demonstrates that dysfunction of this system is associated with pathological protein accumulation, neuroinflammation, vascular impairment, and progressive neurodegeneration. More importantly, an increasing number of preclinical studies indicate that multiple components of the glymphatic–lymphatic axis, including glymphatic transport, meningeal lymphatic drainage, and their upstream regulatory pathways, are amenable to therapeutic modulation. Enhancement of these pathways has been associated with improved CSF drainage, enhanced clearance of pathological proteins, attenuation of neuroinflammation, restoration of CSF dynamics, and amelioration of disease-related phenotypes across diverse animal models. Collectively, these findings provide proof-of-concept that modulation of endogenous brain clearance pathways may represent a biologically plausible therapeutic strategy.

Nevertheless, important questions remain regarding the relative contributions of different clearance pathways, their interactions across distinct anatomical compartments, and their causal roles in human neurological diseases. Although accumulating evidence supports the biological importance of the glymphatic–lymphatic axis, current findings are derived predominantly from experimental models, underscoring the need for rigorous validation in humans.

Taken together, the glymphatic–lymphatic axis may be viewed not only as an anatomical drainage network but also as an integrated physiological framework linking fluid transport, vascular biology, immune regulation, and brain–periphery communication. Rather than replacing existing protein-targeted therapies, interventions targeting this system may ultimately complement them by restoring endogenous brain clearance together with broader neurovascular, ISF, and immune homeostasis. Continued investigation of the glymphatic–lymphatic axis is therefore expected to deepen our understanding of neurodegenerative disease mechanisms while providing new opportunities for translational therapeutic development.

## Data Availability

No datasets were generated or analysed during the current study.

## Abbreviations

Aβ: β-Amyloid
ACE points: Arachnoid cuff exit points
AD: Alzheimer’s disease
AQP4: Aquaporin-4
CNS: Central nervous system
CSF: Cerebrospinal fluid
DTI-ALPS: Diffusion tensor image analysis along the perivascular space
ISF: Interstitial fluid
mLVs: Meningeal lymphatic vessels
PD: Parkinson’s disease
SAS: Subarachnoid space
SLYM: Subarachnoid lymphatic-like membrane
VEGF-C: Vascular endothelial growth factor-C
